# Extraction of active, contaminant degrading enzymes from soil

**DOI:** 10.1016/j.apsoil.2023.104841

**Published:** 2023-02-17

**Authors:** Wambura E. Chacha, Huu-Tuan Tran, William R. Scarlett, Justin M. Hutchison

**Affiliations:** Civil, Environmental, and Architectural Engineering, University of Kansas, 1530 W 15th St., Lawrence, KS 66045, United States of America

**Keywords:** Biodegradation, Intracellular enzymes, Perchlorate reductase, Soil microorganisms, Humic substances

## Abstract

Soil microorganisms play critical roles in the degradation of micro-and nano-pollutants, and the corresponding proteins and enzymes play roles in pollutant recognition, transportation, and degradation. Our ability to study these pathways from soil samples is often complicated by the complex processes involved in extracting proteins from soil matrices. This study aimed to develop a new protein soil extraction protocol that yielded active, intracellular enzymes from the perchlorate degradation pathway, particularly perchlorate reductase. An indirect method, which focused on first separating the cells from the soil matrix, followed by cell lysis and enzyme extraction, was evaluated. The optimized indirect method achieved a final extraction efficiency of the active enzyme and total protein of 15.7 % and 3.3 %, respectively. The final step of separating enzymes from residual soil components resulted in the highest activity and protein losses of 67.7 % ± 14.8 % and 91.8 % ± 1.8 %, respectively. Five buffers, each at different concentrations (0.01 M, 0.05 M, and 0.1 M), were tested to enhance enzyme extraction efficiency. The best extractant requires careful consideration between the highest activity and the quality of the recovered enzymes. Coextraction of humic substances could be minimized by using 0.1 M as compared to 0.01 M and 0.05 M of sodium pyrophosphate; however, this resulted in less recovered activity compared to lower extractant concentrations.

## 1. Introduction

Soil enzymes are primarily sourced from the soil microbial biomass (Tabatabai and Fu, 2021), and broadly speaking, these enzymes can be located extra- or intracellularly (Burns, 1982; Nannipieri, 1994). Soil protein extraction studies began in the 1910s, and most work has focused on extracellular enzymes involved in nutrient cycling and the mechanisms for the formation of organo-mineral complexes in soil (Nannipieri and Smalla, 2006; Fornasier et al., 2011). Thus, many early studies focused on extracellular enzymes, including urease, phosphatases, proteases, and β-glucosidases (Nannipieri, 2006; Nannipieri and Smalla, 2006). Studies evaluating the role of intracellular enzymes are limited as intracellular enzymes can be lost when cells lyse, or intracellular enzymes are not detected in enzyme assay as substrates cannot diffuse into well-protected cells (Duly and Nannipieri, 2011).

Despite efforts at extracting total protein content, there remains a large gap in effectively extracting proteins from the soil matrix. This is especially true of extracting active proteins, which could be used in emerging proteomic analyses such as thermal shift assays (Savitski et al., 2014). However, the majority of studies (Del Pozo et al., 2014) employed denaturing conditions such as boiling (Ogunseitan, 1993; Chourey et al., 2010; Bastida et al., 2014) and denaturing extraction buffers such as sodium dodecyl sulfate (SDS) (Ogunseitan, 1993; Chourey et al., 2010; Keiblinger et al., 2012) and dithiothreitol (Singleton et al., 2003). These methods have been used for downstream metaproteomic analysis from soil environments (Schulze et al., 2005; Wang et al., 2022), functional biomarker analysis of proteins from soil or groundwater (Lopez-Barea and Gomez-Ariza, 2006; Benndorf et al., 2007), stress response analysis of mixed cultures after exposure to toxic compounds (Lacerda et al., 2007), and environmental metallomics analysis (Lopez-Barea and Gomez-Ariza, 2006).

While several studies have examined extracellular enzymes for their importance in nutrient (i.e., carbon and nitrogen) cycling, few studies have examined the extraction of active and intracellular enzymes involved in contaminant reduction. Intracellular proteins include highly specific and specialized enzymes critical for pollutant degradation, including perchlorate, an endocrine-disrupting compound (Hutchison et al., 2013; Hutchison et al., 2017), and novel downstream proteomic processes require active enzymes (Guo et al., 2021). Therefore, this study aimed to propose an indirect method of extracting active enzymes from soil using a model organism, *Azospira oryzae*, a known perchlorate-reducing and soil-relevant bacteria. (Reinhold-Hurek et al., 1993; Hurek et al., 2000). The indirect method first separated cells from soil using sucrose density gradient centrifugation (SDGC), followed by cell lysis and enzyme separation from residual soil components. Active and total protein recovery was determined through perchlorate-reducing activity assays (Kengen et al., 1999; Heinnickel et al., 2011; Hutchison et al., 2013) and the bicinchoninic acid (BCA) assay, respectively. In this study, active protein refers to the protein or enzyme which maintains its catalytically viable structure, whereas total protein refers to the whole proteome regardless if it is active or denatured. The efficiency of this extraction method was evaluated at critical procedural steps to elucidate the impact of overall extraction efficiency. In addition, five extractant buffers (potassium sulfate, potassium citrate, potassium phosphate, sodium hydroxide, and sodium pyrophosphate) at three concentrations (0.01 M, 0.05 M, and 0.1 M) were conducted to enhance the extraction efficiency. These extractants were tested because of their success in extracting proteins or enzymes in a previous study (Greenfield et al., 2018).

## 2. Materials and methods

### 2.1. Chemicals and reagents

Unless otherwise specified, chemicals were purchased from Fisher Scientific (Pittsburgh, PA). Deionized water (18.2 MΩ cm) was purified from Milli-Q (Millipore Milli-Q^®^ Integral 10) Water Purification System and was used to prepare all solutions.

### 2.2. Soil description and preparation

Soil samples were collected from the topsoil (0–10 cm) in West Lawrence in Kansas (38°56′48.3′′ N 95°18′21.9′′ W) in a lawn of a residential area on October 22, 2020. The soil was sieved to pass a 5 mm mesh and stored at 4 °C (Liu et al., 2010). As bacteria were inoculated into the soil at the beginning of the experiments, careful preservation of the natural soil flora was not performed. Soil characterization was performed using approximately 150 g of soil. The soil properties were analyzed (Brown, 1998), including soil organic matter content, clay content, pH, electrical conductivity, and cation exchange capacity, as these factors are known to influence protein adsorption and impact enzyme extraction efficiency (Table 1) (Greenfield et al., 2018). U.S. Geological Survey soil type mapping indicates the sample is a silty clay loam, and the results are typical of this type of soil (Shirazi and Boersma, 1984). According to U.S. Soil Taxonomy, the soil is in the order Mollisols and suborder Udolls.

### 2.3. Preparation of A. oryzae cells

*A. oryzae* strain PS (ATCC number BAA-33) was grown as previously described (Hutchison et al., 2013; Hutchison and Zilles, 2015). Additional details on media preparation and growth curves are provided in the Supplementary Data (Section S.1, Fig. S.1).

### 2.4. Extraction experimental design

**Fig. 1.**
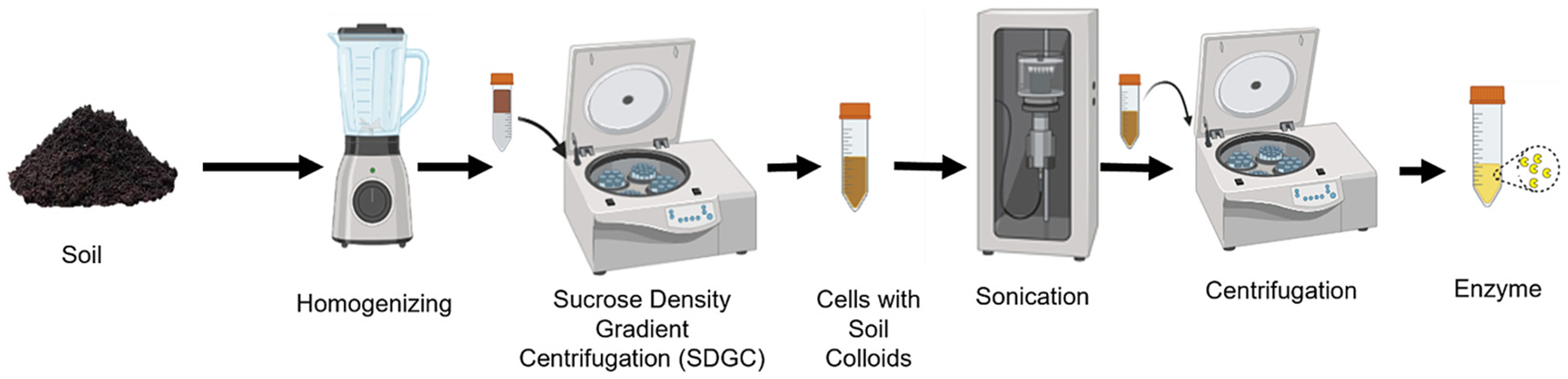
Schematic representation of the indirect extraction method used in this study.

A direct method was tested that involved lysing cells in the soil followed by enzyme separation but failed to recover appreciable enzyme activity (Section S.2, Fig. S.2). In addition, since the direct method required spiking unrealistically high mass of the *A. oryzae* bacterial cells, further analysis with the direct method was discontinued. The indirect extraction method focused on first separating the cells from the soil matrix, followed by cell lysis and enzyme separation from residual soil components (Fig. 1). The indirect extraction steps are described briefly with additional information in the Supplementary Data (Sections S.3–S.6). Soil (20 g) was spiked with either a high (0.5 g) or low (0.1 g) amount of wet mass of *A. oryzae* cells followed by the addition of 100 mL of 0.2 % sodium pyrophosphate solution. Freshly harvested cells were used in all extraction studies (Section S.4). The mixture was homogenized by a blender with a rotation at 22,000 rpm for 15 cycles, where each cycle lasted 6 s with rest intervals of 2 s (Liu et al., 2010). The soil cell homogenate was carefully layered on 100 mL of the sucrose solution. The biphasic layer was centrifuged at 5500 ×*g* for 2 min at 4 °C. The supernatant was recovered. DAPI staining was performed from 500 μL samples taken before and after centrifugation. After centrifugation, cells were diluted with a 0.33 volume of 0.8 % sodium chloride. The solution was centrifuged to pellet the cells at 16,300 ×*g* for 20 min at 4 °C.

Cell pellets were resuspended with a 2 mL volume of 50 mM phosphate buffer (pH 6.0) per gram of the wet pellet mass with 0.1 mg/L DNase. Cells were lysed by sonication (Branson Digital Sonifier 250), using a 1/4′′ sonication tip and an amplitude of 60 % for three lysis cycles of 5 min, alternating between a three-seconds on and 2 s off, followed by 5 min on ice. After sonication, the volume of the lysate was recorded, and approximately 1 mL of the lysate was collected, treated with glycerol to a final concentration of 10 % (*v*/v), and stored in the −80 °C freezer for further enzymatic and protein analysis. The remaining lysate was centrifuged at 5000 ×*g* for 15 min at 4 °C. The supernatant was collected, treated with glycerol to a final concentration of 10 % (*v*/v), and stored in the −80 °C freezer for further enzymatic and protein analysis. The initial activity used in normalizing the recovery efficiency was determined using *A. oryzae* cell lysates not inoculated into soils. These lysates were produced using the same sonication intensity and final centrifugation step. A negative control to ensure the soils did not have perchlorate-reducing activity was performed with uninoculated soils.

Different buffers may impact the protein separation in the final centrifugation step. To test the final step specifically, a clean, uninoculated soil sample was processed up to the final centrifugation step. A 400 μL volume of lysed *A. oryzae* cells was spiked into the processed soil and mixed thoroughly with five different buffers, each at three different concentrations of 0.01 M, 0.05 M, and 0.1 M. The buffers included sodium pyrophosphate, sodium hydroxide, potassium citrate, potassium phosphate buffer, and potassium sulfate. Deionized water was also tested to compare the extraction efficiency with buffers. The lysate and soil extract was incubated at 4 °C for 30 min (Greenfield et al., 2018). One mL of sample was collected. The remaining sample was centrifuged at 18,000 ×*g* for 60 s and the supernatant was collected (Greenfield et al., 2018). The before and after centrifugation samples were treated with glycerol to a final concentration of 10 % (*v*/v) and stored in the −80 °C freezer for further enzymatic and protein analysis. The recovered protein and enzyme activity were compared to tests with *A. oryzae* cell lysates. The extraction efficiency was determined based on the theoretical amount of protein and enzyme activity added and the final protein and enzyme activity determined after centrifugation. The efficiency accounts for changes in activity due to enzyme exposure to a new buffer (e.g., impacts of high pH buffer on the activity) and the losses associated with separation from the soil. All tests were performed in triplicate, which included independent cell cultures.

### 2.5. Protein mass, enzyme activity determination, and relative quality of extracted samples

Protein quantification was analyzed using the colorimetric micro-plate BCA assay. Dilutions (5, 10, or 15 factors) were performed in the respective buffers. The protein for mass balance principles was reported as a mass of protein per gram of cell. Effects of soil matrix on the BCA protein assay were determined (Section S.7, Table S.2). Protein concentration measurements were performed with analytical duplicates.

Perchlorate-reducing enzyme activity was determined using the colorimetric methyl viologen (MV) at room temperature (22 °C) as previously described (Kengen et al., 1999) using anaerobic cuvettes with cap and septa in a COY anoxic chamber. Absorbance was recorded at 578 nm. Activity (Units (U), defined as 1 μmole of MV oxidized per minute) was calculated using an extinction coefficient of 13.1 mM^−1^ cm^−1^ (Eq. (S.1)). The reported activity value was obtained by subtracting the background activity (oxygen or lysate reactions) from the perchlorate activity. Activity measurements were performed in analytical duplicates.

The spectrophotometric ratio (254 nm/400 nm) was used to evaluate the sample humification and relative quality (Carter et al., 2012; Peacock et al., 2014; Greenfield et al., 2018). The absorbance of the extracted sample was measured in a quartz cuvette (9-Q-10-GL14-S, Starna Cells, Atascadero, CA). The sample with the highest ratio was used to normalize the relative quality of the other measurements.

### 2.6. Statistical analysis

The assumption of equal normality was tested using Shapiro-Wilk test. Statistical analysis was performed using the independent-samples *t*-test when comparing two data sets. ANOVA on ranks was performed when comparing more than two data sets with Tukey’s comparison. Samples were considered significantly different when the two-tailed *P* value was less than alpha (0.05).

## 3. Results

### 3.1. Extraction efficiency of the enzyme from the soil by the indirect method

**Fig. 2.**
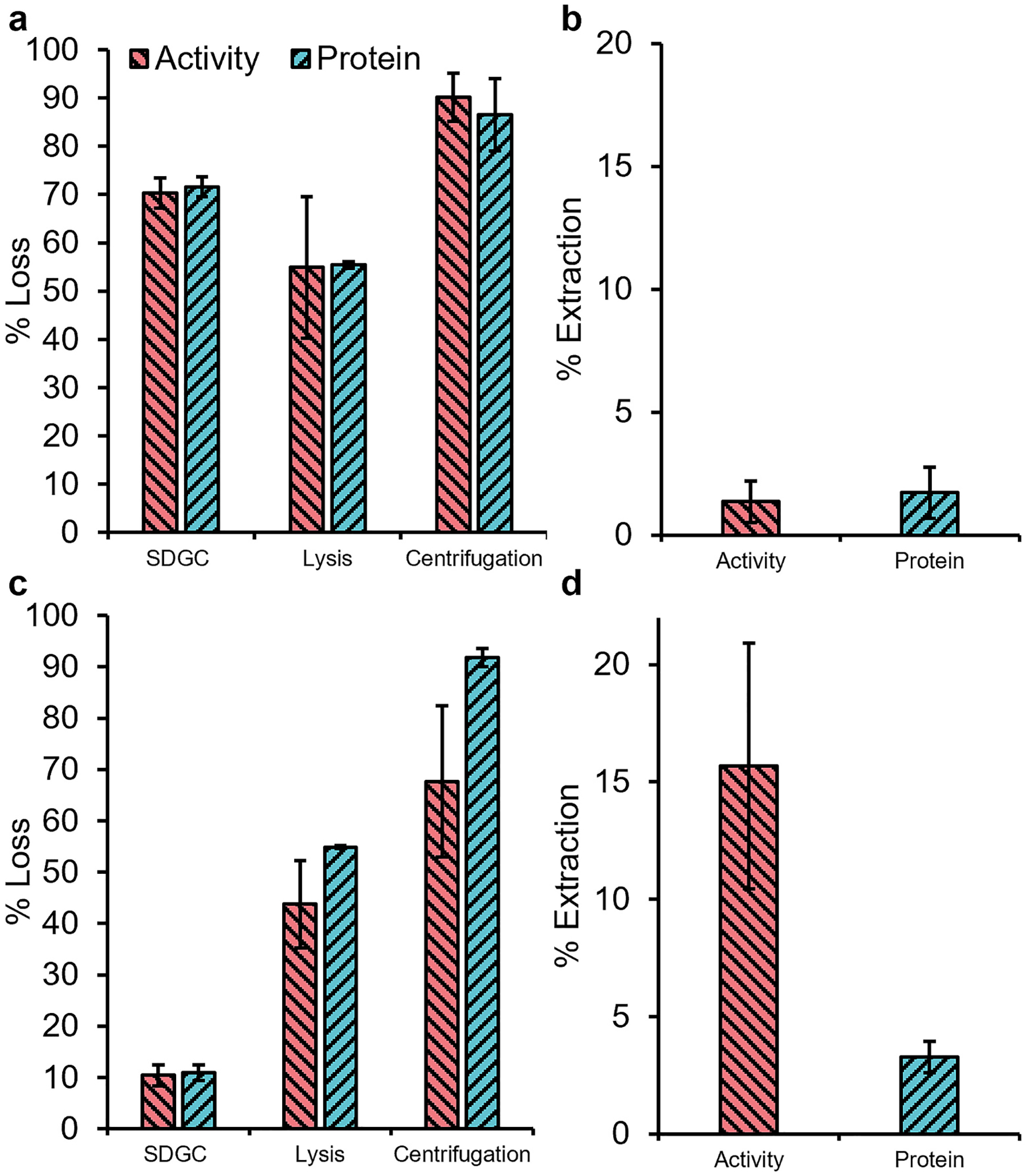
a) Activity (red) and protein (blue) losses when the soil is inoculated with higher cell mass for the indirect extraction process and using an unoptimized extraction protocol. b) The final extraction percentage for the high cell mass loading. c) Activity and protein losses when the soil is inoculated with a realistic cell mass (≤0.1g_cell_ 20 ${\text{g}}_{\text{soil}}^{-1}$), representative of cell-soil ratio and d) final activity and protein recovery. Experiments were performed with biological triplicates and measurements in duplicate. Error bars are standard deviations.

The overall activity and protein extracted in the indirect method were 1.4 % ± 0.9 % and 1.7 % ± 1.0 % (Fig. 2), respectively. As an unrealistic cell loading mass (0.5 g_cells_ (20 g_soil_)^−1^) could have exceeded the method recovery capacity, the amount of *A. oryzae* wet cell mass was reduced to 0.1 g_cells_ (20 g_soil_)^−1^, which is more realistic of environmental samples (Liu et al., 2010). To reflect the recovery more accurately from the SDGC, DAPI staining was implemented (Fig. S.4). This imaging better reflected the efficiency of the SDGC procedural step, with up to 90 % of cells recovered (total protein and activity losses of 10.94 % ± 1.49 % and 10.43 % ± 2.09 %, respectively) (Fig. 2).

Following optimization of the sonication protocol (Fig. S.5), activity recovery improved by a further 10 %. However, high losses were still observed in the final centrifugation step. Nonetheless, the overall activity extraction efficiency after the modifications significantly improved to 15.7 % ± 5.2 % (*p* = 0.021) versus the initial recovery of 1.4 % ± 0.9 % (Fig. 2). This contrasts with the total protein recovery, which did not have a statistically significant difference (3.3 % ± 0.6 % from 1.7 % ± 1.0 %, *p* = 0.380).

### 3.2. Extraction efficiency and protein purity using different extractants at different concentrations

**Fig. 3.**
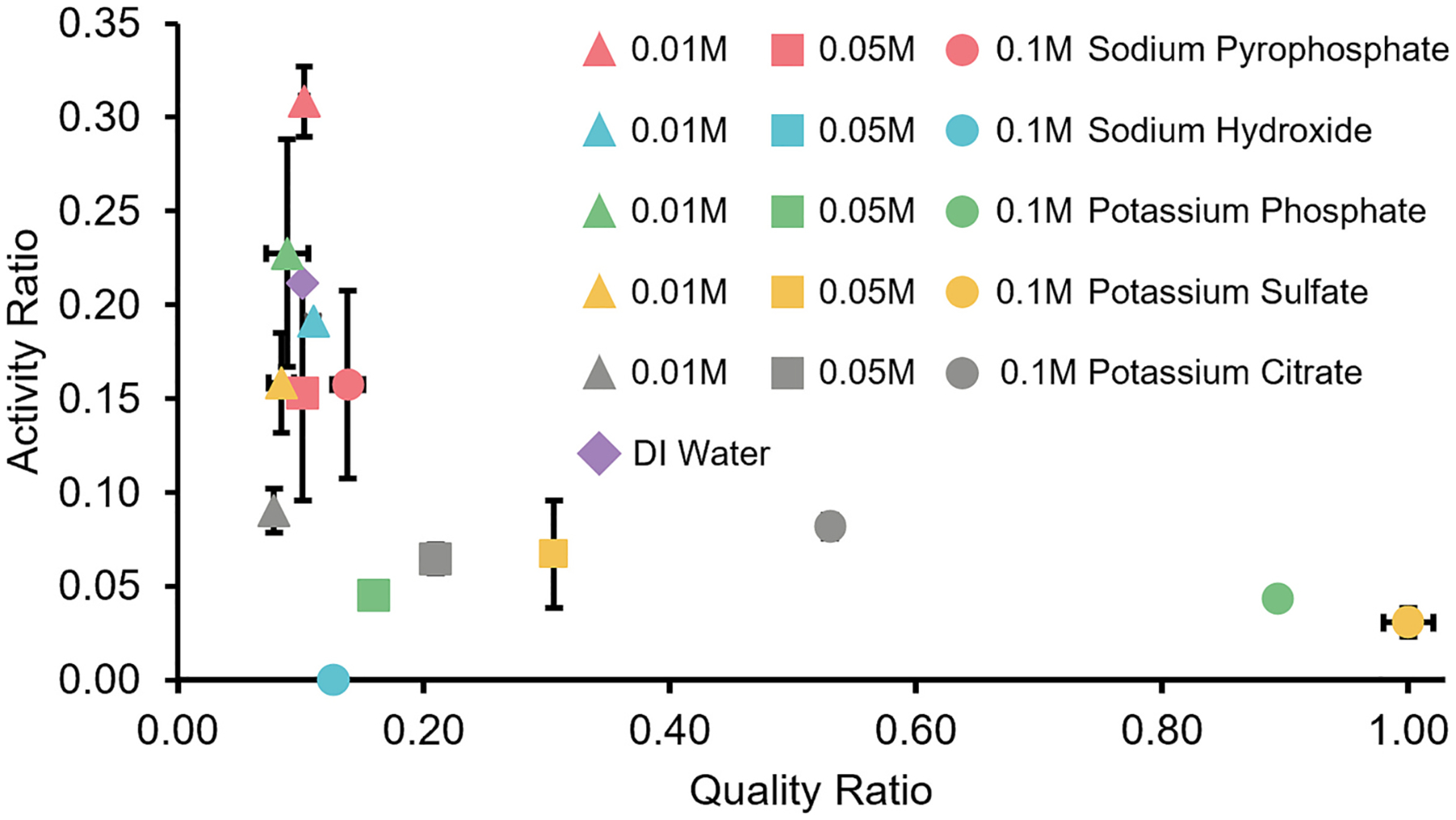
Average enzyme activity and sample quality ratios for samples recovered at different concentrations of the extractants. The activity ratio was determined based on the recovered enzyme activity (U ${\text{g}}_{\text{cells}}^{-1}$) divided by the original activity. The quality ratio was a relative comparison between the samples’ 254 nm/400 nm ratio divided by the highest 254 nm/400 nm ratio sample (0.1 M potassium sulfate). The 0.05 M and 0.1 M sodium hydroxide sample points overlap. Experiment was performed with biological triplicates and measurements in duplicate. Error bars are standard deviations with some errors too small to visualize on the graph.

To further improve the final centrifugation step, five different extractant buffers were tested (Bremner, 1949; Nannipieri et al., 1974; Haney et al., 2001; Friedel and Scheller, 2002; Masciandaro et al., 2008; Greenfield et al., 2018): potassium sulfate, potassium citrate, potassium phosphate, sodium hydroxide, and sodium pyrophosphate. Three buffer concentrations (0.01 M, 0.05 M, and 0.1 M) were tested, and the solution pH was recorded (Table S.3). Water as an extractant was also tested. Generally, the enzyme activity decreases with the increase in the concentration of the extracting solutions (Figs. 3, S.6) except for potassium citrate concentrations 0.05 M to 0.1 M. Enzymes extracted with sodium pyrophosphate (0.01 M) retained the highest activity of 31.4 % ± 1.9 %.

However, the final enzymes extracted using different buffers had distinct color differences, indicating coextraction of humic substances (Fig. S.7). When the extractant concentrations were 0.01 M, the supernatants were dark in color, indicating that high humic compounds were coextracted for all the extractants (Fig. S.7a). At a concentration of 0.05 M, the supernatants from sodium hydroxide and sodium pyrophosphate as well as deionized water were darker than for potassium sulfate, potassium phosphate, and potassium citrate (Fig. S.7b) likely due to higher concentrations of coextracted humic substances. A similar outcome was observed for 0.1 M extractant solutions (Fig. S.7c). The visual distinct differences of the final extractions were well complemented with UV spectrophotometric measurements (Table S.4). The ratio (254 nm/400 nm) provides a measure of humification (Carter et al., 2012; Peacock et al., 2014; Greenfield et al., 2018), where a higher ratio indicates improved sample quality (i.e., low humic substance contamination) (Graham et al., 2012). The quality of the sample improved with increasing extractant concentration. Ultimately, a tradeoff between reduced coextracted humic substances and increased enzyme recovery was observed. For example, potassium sulfate, potassium phosphate, and potassium citrate at 0.1 M concentrations achieved the highest purity but only yielded <8.2 % enzyme activity (Fig. 3).

## 4. Discussion

The indirect enzyme extraction method and the efficiency at specific procedural points were determined for soils inoculated with *A. oryzae* cells or lysates. The enzyme quality was assessed for the presence of coextracted humic substances, which could interfere with downstream activity-based profiling (Guo et al., 2021) or advanced mass spectro-scopic methods, including thermal shift assays (Jafari et al., 2014; Savitski et al., 2014). Here, we compare our extraction efficiencies for enzyme activity and overall protein quality to other efforts in the literature and discuss other quality considerations when using soil extracted, active enzymes in downstream analysis.

The indirect extraction method, consisting of SDGC separation, lysis by sonication, and centrifugation, demonstrated promising results for active, intracellular protein recovery. The SDGC separation procedural step realized similar cell recoveries to a previous study (70 %–90 %) (Liu et al., 2010). Our lysis recovery using sonication was less efficient than in previous studies (85 %); however, that study used a different cell type (HT-29) and only tracked protein content and not enzyme activity (Myers et al., 2011).

The last procedural step of the indirect method, centrifugation, resulted in the highest losses (Fig. 2), which could have been caused by adsorption of the enzymes to coextracted humic substances and soil colloids (O’Melia, 1969) through ion exchange, H-bond, electrostatic attraction, Van der Waals, or complexion interactions. To improve the final recovery, different extraction buffers at different concentrations were tested in the last procedural recovery step (the centrifugation step) of the indirect method. The highest perchlorate-reducing activity recovery efficiency (31.4 % ± 1.9 %) was obtained using 0.01 M sodium pyrophosphate. These recovery results fall within ranges reported in the literature for 0.01 M sodium pyrophosphate where urease extracted from podzol soil ranged (30 %–40 %) (Nannipieri et al., 1974). Comparable studies using water had recoveries of 10–60 % (Greenfield et al., 2018) and using 0.01 M sodium hydroxide had recoveries of 25 %–74 % (Greenfield et al., 2018). Our results for different concentrations of potassium citrate, potassium phosphate, and potassium sulfate were comparable to previously published reports where the range of values were 13 %–63 %, 13 %–45 %, and 11 %–34 % (Masciandaro et al., 2008), respectively. The observation of the decreasing trend in active enzyme extraction efficiency with increasing buffer concentration is consistent with previous studies (Masciandaro et al., 2008; Greenfield et al., 2018). Similarly, the use of higher concentrations extractants and the corresponding higher ionic strength extract less humic substances compared to lower concentrations (Kipton et al., 1992).

One driving factor that could explain the observed trends with the buffers is pH. The perchlorate-reducing enzymes have basic isoelectric points (pI) (perchlorate reductase: pI 9.1 (Steinberg et al., 2005) and chlorite dismutase: pI 9.6 (Streit and DuBois, 2008)). Extraction buffers with pH less than the isoelectric point could result in an overall net positive charge of the enzymes and an increased possibility of binding to negatively charged soil colloids. The results show that the extractant which had the highest enzyme activity was 0.01 M sodium pyrophosphate (pH = 9.66). A previous study showed robust perchlorate reduction over the pH range 6–9 (Hutchison and Zilles, 2015). While sodium hydroxide extractants had higher pH, this is likely outside of the acceptable range for the perchlorate-reducing enzymes, especially for the 0.05 M and 0.1 M concentrations, where no activity was detected. In addition, the highest enzyme activity was found when sodium pyrophosphate concentration was 0.01 M (pH = 9.66), while sodium pyrophosphate concentrations of 0.05 M (pH = 9.60) and 0.1 M (pH = 9.61) had lower enzyme activity, which may have been caused by the salting out effect due to the increased sodium pyrophosphate concentration. It was determined in a previous study (Shih et al., 1992) that the higher the salt concentration, the higher the salting out effect.

While 0.01 M sodium pyrophosphate had the highest active perchlorate-reducing enzyme recovery, other considerations, including the coextraction of humic substances must also be considered. The coextraction of humic substances can interfere with downstream imaging (SDS-PAGE gel, Fig. S.8, (Murase et al., 2003)) and proteomic and mass spectrometry pipelines (Matsumoto et al., 2000; Qian and Hettich, 2017). Changes in ionic strength could explain part of the impact on the coextraction of humic substances with higher ionic strength promoting coagulation and precipitation occurring at high pH (e.g., sodium hydroxide (Qian and Hettich, 2017)) or the ability of potassium salts to induce conformational changes in the humic substances and protein structures (Shih et al., 1992). However, this process of coagulation and precipitation could also result in the capture and loss of the target enzyme.

Overall, our protein recovery corresponded to 3.3 % ± 0.6 % and 15.7 % ± 5.2 % of the initial protein content and activity loaded onto the soil, respectively. While our protein losses are high compared to previous reports, which obtained a protein recovery efficiency of 62 %–83 % (Kanerva et al., 2013) and 75 %–85 % (Criquet et al., 2002), those studies used BSA and tracked protein concentration but not enzyme activity. In addition, our total intracellular protein recovered (0.12 μg (g_soil_)^−1^) was generally lower than studies focused on total protein extraction, which ranged from 0.03 to 30.48 μg (g_soil_)^−1^ (Bastida et al., 2018). The lower mass of protein recovered is likely explained by our study’s focus on intracellular proteins.

Results on overall activity recovery from previous studies have a wide range (e.g., 0.1 %–0.28 % for extracellular enzymes (Bastida et al., 2018), 0.1 %–5.2 % for arylsulphatase and phosphodiesterase enzymes (Vepsäläinen, 2001), 11 %–36 % for protease activity (Bonmati et al., 1998), and ≤1 % for different functional protein groups (Benndorf et al., 2007)). Similarly, our results showed a wide range of recovery based on different methodological approaches and buffers suggesting that careful consideration of the procedure is required to achieve the intended protein extraction goal.

## 5. Conclusions

This work demonstrated the potential for extracting active perchlorate-reducing enzymes from soil matrix using our indirect method. The method consisted of three steps, and the losses at each step were quantified to identify barriers to effective active protein extraction from soil. The centrifugation step separating soluble enzymes from humic substances contributed a majority of the losses of total protein and active enzyme; however, these losses could be minimized using 0.01 M sodium pyrophosphate. These results compare favorably to previous protein and enzyme extraction protocols where the studies relied on soil protein surrogates, extracted denatured proteins, and other extracellular enzymes. In this study, one soil (silty clay loam) was tested in the extraction procedure. However, it is important to note that different soil characteristics, such as soil organic matter content, clay content, pH, electrical conductivity, and cation exchange capacity, could impede enzyme extraction efficiency due to adsorption on the coextracted humic substances.

While total protein recovered was low (3.3 %), our specialized extraction protocol recovered a higher percent of the target enzyme, perchlorate reductase, (15.7 %). This indicates that further studies are needed to understand the complex relationships between the diverse sets of soil and protein properties to optimize extraction protocol(s) to facilitate holistic proteomic approaches. We have identified three critical steps for the extraction of intracellular enzymes, including cell separation, cell lysis, and enzyme extraction. Alternative approaches could include using a Nycodenz solution for cell extraction; other mechanical or chemical techniques for cell lysis; and precipitation, filtration, aqueous two phase or three phase separation systems for enzyme extraction. Dependent on the properties of the enzyme and soil, unique combinations of these techniques may be required to achieve efficient extraction of the target protein.

## Supplementary Material

Supplemental Material

## Figures and Tables

**Table 1:** Characteristics of the soil with averages and standard deviations provided.

Parameters	Values
pH	6.55 ± 0.05
Electrical conductivity (dS/m)	2.29 ± 0.03
Total organic carbon (%)	2.29 ± 0.13
Total nitrogen (%)	0.20 ± 0.01
Sand (%)	13 ± 5
Silt (%)	53 ± 3
Clay (%)	34 ± 2
Cation exchange capacity (meq/100 g)	26.2 ± 0.9
Calcium (ppm)	3420 ± 44
Magnesium (ppm)	442 ± 11
Potassium (ppm)	288.7 ± 1.7
Sodium (ppm)	226 ± 34

## Data Availability

Data will be made available on request.
