# Supplementary material for: Extraction of active, contaminant degrading enzymes from soil
Source: Appl Soil Ecol. Author manuscript; Available in PMC 2023 Oct 2. (PMC10544838; doi:10.1016/j.apsoil.2023.104841)
Supplement: Supplemental Material [file NIHMS1878448-supplement-Supplemental_Material.docx]

*Supplementary Data*

Extraction of active, contaminant-degrading enzymes from soil

Wambura E. Chacha^1^, Huu-Tuan Tran^1^, William R. Scarlett^1^, and Justin M. Hutchison^1*^

^1^ Civil, Environmental, and Architectural Engineering

University of Kansas

1530 W 15th St.

Lawrence, KS 66045

^*^ corresponding author contact information: Email Address: [jhutch@ku.edu](mailto:jhutch@ku.edu), Phone: 785-864-6170

Figures – 8

Tables – 4

Equations – 1

Contents

Contents 2

S.1 Growth of the *Azospira oryzae* cells 3

S.2 Direct Extraction 4

S.3 Determination of the sucrose solution density 6

S.4 The use of fresh cells versus frozen cells 6

S.5 DAPI staining, microscopy and cell enumeration using Cellprofiler 7

S.6 BCA accuracy in the presence of soil extracts 10

S.7 Enzyme activity calculation 10

S.8 Sonication optimization 11

S.9 Enzyme activity and co-extraction of humic substances 13

References 17

# S.1 Growth of the *Azospira oryzae* cells

Aerobic cultures were grown using LB-Lennox agar and liquid media. The transition to anaerobic growth was performed using a modified Ralph S. Tanner (RST) media at 150 rpm at 30°C (Thermo Scientific Max Q 6000) for 48 hours until the cells reached an optical density (OD_600_) of approximately 0.4 (Hutchison et al., 2013). The *A. oryzae* substrate-growth curve followed an expected pattern with an increase in optical density corresponding to a decrease in perchlorate concentrations (Figure S.1). The anaerobic growth was used to make 10 mL culture stocks with 10% glycerol stored at -80°C.

Five mL of culture stock were used to inoculate one liter of anaerobic RST media. Cells were prepared in four-liter batches in RST media. The inoculated one-liter cell cultures were incubated at 150 rpm at 30°C for approximately 48 hours until the culture reached an OD_600_ of approximately 0.6. Cells were pelleted at 3300 xg for 15 minutes at 4°C (Sorvall^TM^ Legend^TM^ XT/XF Centrifuge). The obtained cells were resuspended and washed with a 50 mM sodium phosphate buffer (PB) at pH 6. The cells were pelleted again at 4050 xg for 15 minutes at 4°C, buffer removed, and the wet mass of the cells was recorded. Cells used in soil enzyme-extraction experiments were immediately spiked into the soil to proceed with the extraction experiments. Fresh cells were used to minimize the disruption of the cell integrity associated with -80°C freezing.

The growth of *A. oryzae* was monitored spectrophotometrically and coincided with a decrease in perchlorate concentration (Figure S.1). Perchlorate was measured using ion chromatography as previously described (Hutchison et al., 2013). The organism reached stationary growth 42 hours after inoculation, which allowed sufficient wet mass of the *A. oryzae* cells to be produced for running the extraction efficiency analysis.


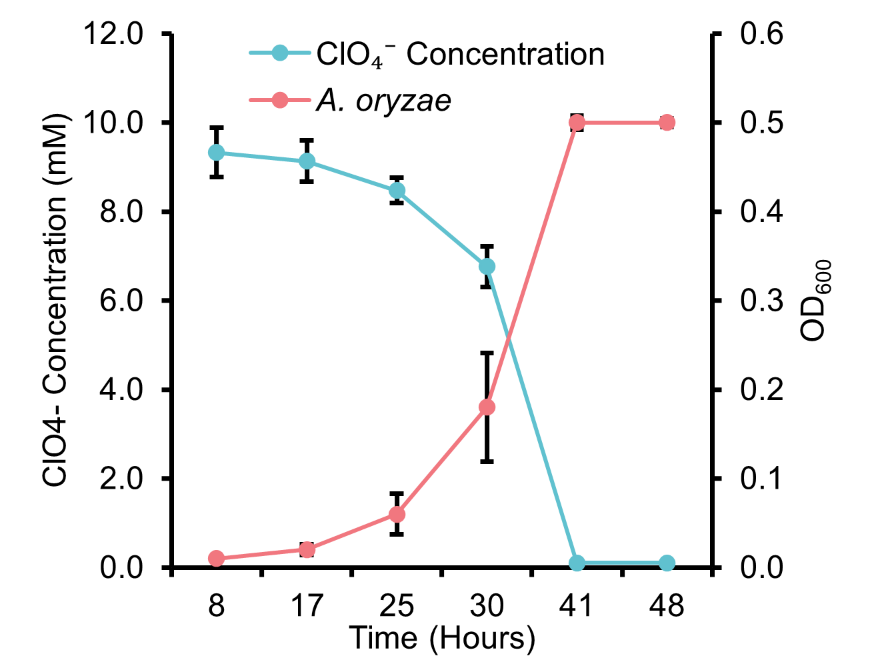


Figure S.1: Growth of *A. oryzae* coincided with decreasing perchlorate concentration. Spectrophotometric measurements were taken at 600 nm. Experiments were performed with biological triplicates. Error bars represent standard deviation.

S.2 Direct extraction

The direct method extracted enzyme content directly from the soil by lysing the cells within the soil matrix followed by separation. The Novipure soil protein kit was carried out based on the manufacturer protocol with modifications. To extract active enzymes, the kit SP1 and SP2 solutions were chilled to 4°C before use. The DTT was kept in the -20°C freezer. An average of 1.94g of *A. oryzae* cells was loaded into 5g of soil, and 15 mL of SP1 solution was added into the 50 mL Novipure bead tube. The tube was vortexed thoroughly and incubated on ice for 10 minutes. The bead beating process was performed using a vortex adapter in the cold room (4°C) and vortexed at a maximum speed of 3200rpm for 10 minutes. After bead beating, the tube was centrifuged for 30 seconds at 4500xg and 4°C to remove the residual soil, and beads from the top of the tube. SP2 solution of 1.5 mL was added to the tube, and the tube was vortexed followed by incubation on ice for 30 minutes. After incubation, the tube was centrifuged at 4500xg for 10 minutes at 4°C to separate the soil particles and the beads from the soluble protein in the supernatant. The supernatant was pipetted to a new tube, and precipitation was performed using an equal volume of 60% w/v (NH_4_)_2_SO_4_, while the pH was adjusted to 7 using 1M KH_2_PO_4_. This was followed by incubation in a 4°C refrigerator for 6 hours (Del Pozo et al., 2014). The protein precipitates were recovered by centrifuging at 14000xg for 25 minutes and resuspended in 50mM PB. As a control, the effects of the direct extraction methods were tested against pure cell lysates (e.g., no soil was included in the sample). Extractions were performed with biological triplicates.

A modified Novipure protein extraction protocol with ammonium precipitation was used. Despite the manufacturer suggested modifications that removed chemicals and physical processes that could denature enzymes, minimal enzyme activity was recovered compared to the initial indirect method tests. These results are not exceptionally surprising given that the kit was originally intended for denatured protein extraction. Nonetheless, other researchers have used this protocol to provide comparable results compared to traditional, denaturing extraction methods (Tartaglia et al., 2020). Further, our use of the ammonium sulfate to precipitate and recover proteins was not effective, with a recovery efficiency of 12.1% ± 2.6%. (Figure S.2a)

The enzyme and protein extraction efficiency were determined using cells without soil for both the indirect and direct extraction methods (Figure S.2b). The two methods had statistically similar total protein content extracted per gram of cell (*p*=0.593); however, the indirect method retained detectable levels of enzyme activity.

Figure S.2: **a)** Enzyme activity (red) and protein content (blue) measured from the Novipure extraction method with ammonium precipitation. **b)** A comparison of the direct and indirect extraction methods on cells without soil.


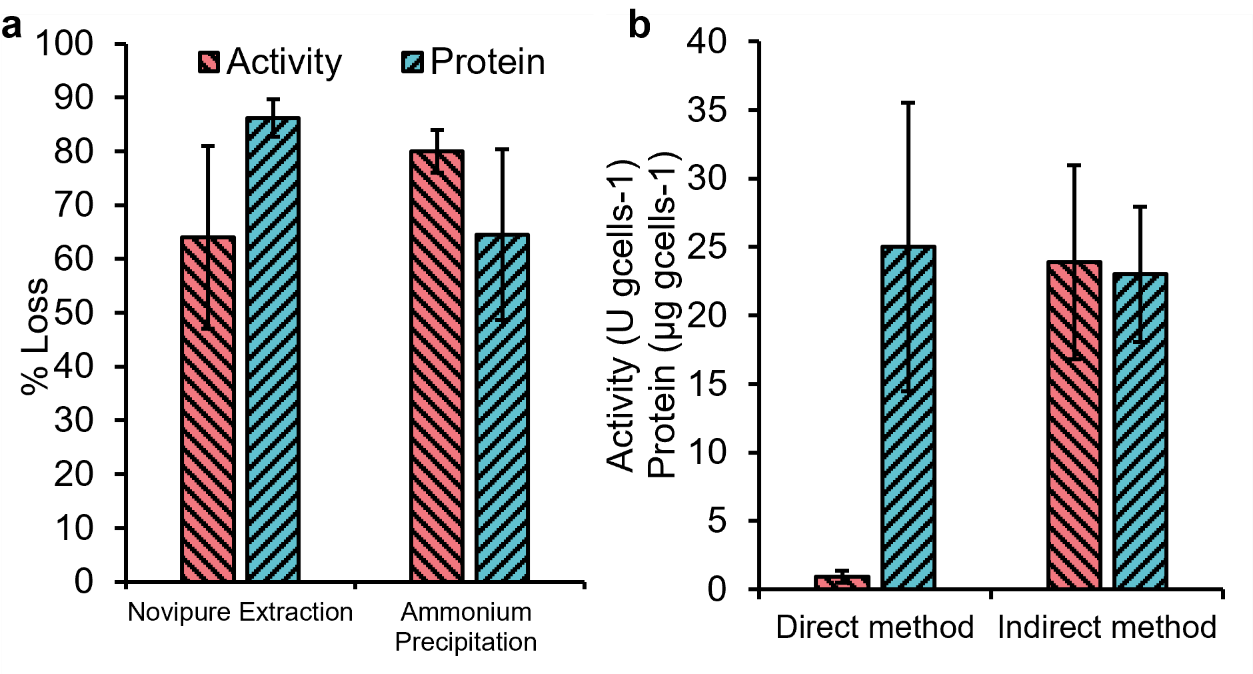


S.3 Determination of the sucrose solution density

The density of a 1.33g mL^-1^ sucrose solution was 1.308 g mL^-1^ (Table S.1). The solution was used to separate bacterial cells (approximate density of 1.1g/ml) from soil particles (approximate density of 2.65g/ml).

Table S.1:Density of the sucrose solution

| Mass, Empty pipette tip (g) | 0.911 |
| --- | --- |
| Mass, Pipette tip with 500ul sucrose (g) | 1.565 |
| Mass of sucrose solution (g) | 0.654 |
| The density of sucrose solution (g/ml) | 1.308 |

# S.4 The use of fresh cells versus frozen cells

The process of storing *A. oryzae* cells by freezing them at -80^0^C has proven to be a viable method for studies that required the extraction of the periplasmic perchlorate reductase enzyme (Hutchison et al., 2013; Hutchison and Zilles, 2015; Hutchison et al., 2017; Hutchison and Zilles, 2018). However, several studies (Storey and Storey, 1988; Wolkers et al., 2007) have shown that freezing and thawing of bacteria result in stress on the cell membrane, which may result in the loss of membrane integrity and may cause the cell contents to be released. In this study, especially for the indirect method, the cell integrity of the loaded cells is of utmost importance to prevent enzyme loss associated with premature cell lysis. To test whether frozen cells would result in the loss of active enzymes during the enzyme extraction from soil, two sucrose density gradient centrifugation (SDGC) extraction experiments were performed with frozen or freshly harvested (non-frozen) cells. For each experiment, a wet cell mass of 1.6 g 20 g^-1^ soil was added. Cell and activity were recovered using methods described in the main manuscript. The results showed that higher enzyme activity was extracted from the experiment with non-frozen cells. (Figure S.3). All experimental data presented in the manuscript were obtained using non-frozen cells.

Figure S.3: Activity comparison of frozen versus non-frozen cells extracted from soil. Experiments were performed with biological replicates and measurements in triplicates. Error bars represent standard deviation.


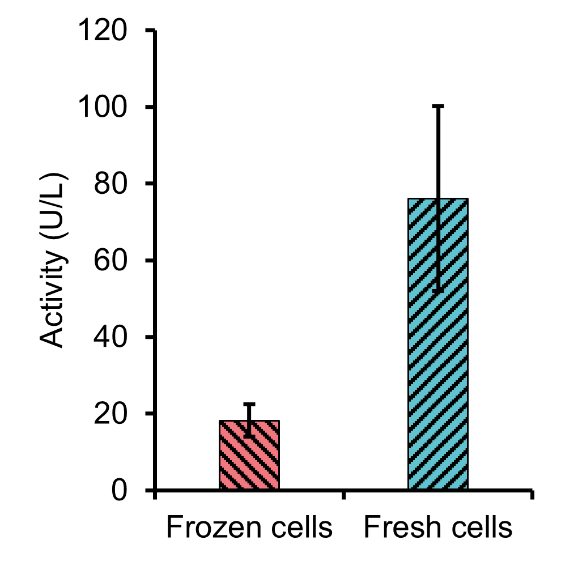


# S.5 DAPI staining, microscopy and cell enumeration using Cellprofiler

DAPI staining was used to determine the extraction efficiency of the cells in the SDGC extraction procedure and followed previously published protocols (van Loosdrecht et al., 2016). Briefly, the samples were collected before and after SDGC extraction. The samples were fixed by adding an equal amount of 4% paraformaldehyde (PFA) and incubated for 2 hours with inversion every 30 minutes. The samples were centrifuged at 3500xg for 8 minutes at 4°C and decanted. The cell pellet was resuspended in the 1x phosphate buffer saline (PBS) (0.03M NaH_2_PO_4_, 0.03M Na_2_HPO_4_, and 0.13M NaCl) and centrifuged again. The pellet was washed twice more with a 1:1 volume of PBS and 95% ethanol solution. The pellets were finally resuspended in a 4°C PBS buffer for later use in the DAPI staining processes. Cell suspension (30 µL) was flame fixed on a microscope slide and washed dropwise using PBS. The slide was incubated in PBS for 3 minutes. DAPI solution was prepared per the manufacturer’s recommendation, and the slide was incubated for 30 minutes in the dark. The slide was washed with PBS and incubated for 3 minutes with PBS. Mounting media (50 µL, VECTASHIELD® Antifade Mounting Medium with DAPI, Vector Laboratories) was added to the slides.

Images were acquired with an epifluorescence inverted microscope (Olympus IX-81 inverted microscope) equipped with dual excitation sources, a lambda LS Xenon 300W and a White LED coupled to 3 automated lambda 10-3 filter wheels. Images were captured with a 100x magnification objective with the imaging program Slidebook 6(6.0.22(36972)x64). Images were exported to the Cellprofiler program (4.1.3) for cell enumeration. As a control, DAPI staining of unloaded soil samples before and after SDGC extraction was performed.

DAPI staining was used for cell enumeration (Liu et al., 2010) and used to estimate the extraction efficiencies of the cells in the SDGC extraction procedural step. Cellprofiler program (4.1.3) was used to enumerate the cells. This was done by importing the images produced by the imaging program Slidebook 6(6.0.22(36972)x64. Under the metadata command, the toggle box beside extract meta data was selected, and the desired Regex name was selected. Names to assign these images was selected as DNA, and all other setting were left as default. For advanced settings, an adaptive threshold was used with a robust background.

Images were captured for the loaded (Figure S.4a and b) and unloaded samples (Figure S.4c and d) before and after centrifugation. The cell count of the loaded sample, before (670.8±77.9), and after SDGC (596.4±59.2), was greater than the unloaded negative control sample, before (100.3±10.3) and after (6.25±4.00). The extraction efficiency was calculated based on the enumerated cells before (670.8±77.9) and after (596.4±59.2) SDGC (Figure S.4 a and b), with an extraction efficiency of 89.1±1.5%. Images of unspiked soil samples before and after SDGC were also collected (Figure S.4 c and d). The extraction efficiency of cells was used to approximate the amount of activity and total protein lost at the SDGC procedural step based determined original *A. oryzae* activity and total protein loaded into the soil.


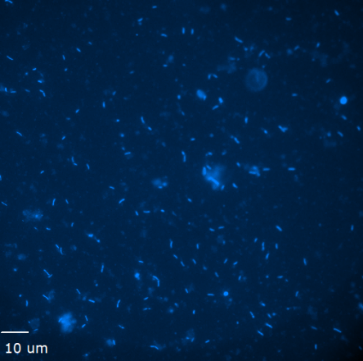

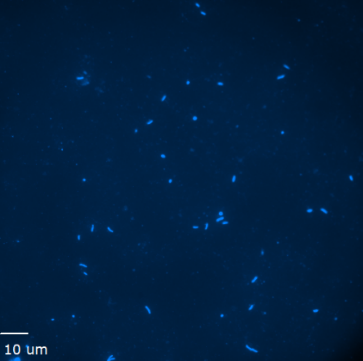

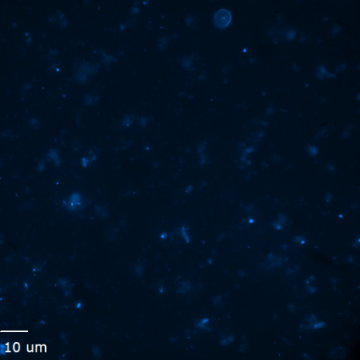

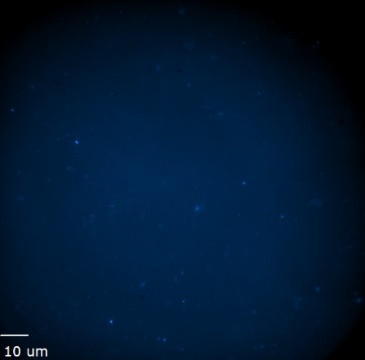


c)

d)

a)

b)

Figure S.4 The DAPI images as obtained from the epifluorescence microscope with (a) the loaded sample before SDGC, (b) the loaded sample after SDGC, (c) the unloaded sample before SDGC, and (d) the unloaded sample after SDGC.

# S.6 Sonication optimization

Sonication was used to lyse cells. Cell resistance to lysis is based on the characteristics of the cell membrane. Previous studies employed a sonication amplitude of 30% to lyse *A. oryzae* cells (Hutchison et al., 2013; Hutchison and Zilles, 2015, 2018). However, in this study, by systematically varying the sonication amplitude and comparing the activity of the enzyme after sonication and after a final centrifugation step (10,000 xg for 15 min). The activity after sonication (before centrifugation) is the activity of the crude lysate consisting of enzymes and other cellular components. Insufficient lysis and separation of the enzymes from these cellular components result in enzyme loss after centrifugation. Selecting the correct amplitude is a balance between sufficient power to lyse cells and too much power that denatures the enzymes. The optimum sonication amplitude to lyse and extract the associated enzymes was 60% (Figure S.5).

Figure S.5: Optimization of the sonication amplitude Sonication amplitudes above 60% resulted in denaturing of the enzyme, while sonication amplitudes below 60% resulted in inefficient lysis and subsequent enzyme extraction from cells. Errors bars are standard deviation of biological triplicate measurements.


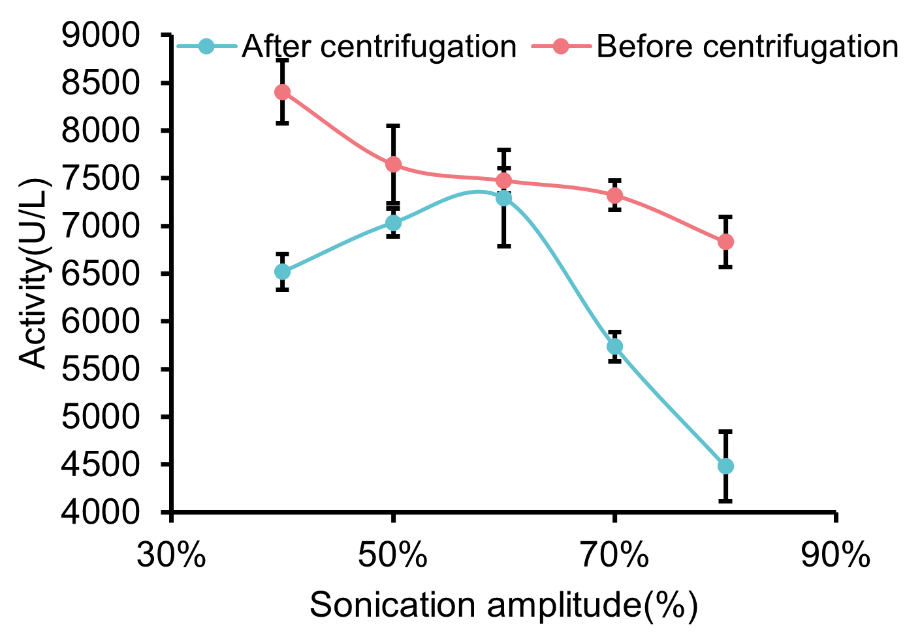


# S.7 BCA accuracy in the presence of soil extracts

To account for variations in BCA measurements due to the presence of soil, BSA protein (100 µg) was spiked into soil. The measured protein difference was compared to the expected protein added. The results show that there is a consistent overestimation of quantified protein by approximately (23 ± 5)% in the presence of humic substances (Table S.2). These results were accounted for in our reporting of protein mass throughout the manuscript.

Table S.2:Determination of the effect of BCA assay on protein quantification

|  | Replicates | | | | | Avg ± Std Dev |
| --- | --- | --- | --- | --- | --- | --- |
| Average protein Concentration (µg ml^-1^) (spiked) | 189 | 140 | 130 | 127 | 133 | 144 ± 26 |
| Average protein Concentration (µg ml^-1^) (unspiked) | 57 | 20 | 11 | 9 | 8 | 21 ± 21 |
| Protein mass difference (µg) | 131 | 120 | 119 | 118 | 125 | 123 ± 5 |
| Expected difference (µg) | 100 | 100 | 100 | 100 | 100 | 100 ± 0 |
| Protein overestimation (%) | 31 | 20 | 20 | 20 | 25 | 23 ± 5 |

# S.8 Enzyme activity calculation

Enzymatic activity was calculated from the slope taken from the colorimetric methyl viologen (MV) assays and Equation S.1(McClatchey, 2002)

$Activity\left( \frac{U}{L} \right)=\frac{\frac{AU}{min}*TV}{\varepsilon*b*SV}*1000$

Equation S.1: Enzyme Activity. The activity of the extract is defined as 1 µmole of reduced MV oxidized per minute, per liter of extract. The change in the slope of the absorbance with respect to minutes (AU/min), the total volume (TV) in µL, the µmolar absorptivity (ε) of MV with a value of 13.1 L/(mMol*cm) as previously determine (Thorneley, 1974), the path length (b) in centimeters (1 cm), and the sample volume (SV) in µL were used to determine activity.

# S.9 Enzyme activity and co-extraction of humic substances

The extraction efficiency of many organic compounds in soil highly depends on pH (Greenfield et al., 2018). Proteins carry a pH-dependent charge which can affect their binding to soil particles and, therefore, have an impact on their recovery from the soil. The pH of the extracting solutions is shown in Table S.3

Table S.3: The pH of the extracting solutions

| Extractant | Concentration | pH |
| --- | --- | --- |
| Deionized water | 0.00 | 6.25 |
| Potassium phosphate  (K_2_HPO_4_, KH_2_PO_4_) | 0.01 | 8.06 |
|  | 0.05 | 8.12 |
|  | 0.10 | 8.02 |
| Potassium citrate  (C_6_H_5_K_3_O_7_) | 0.01 | 7.91 |
|  | 0.05 | 7.87 |
|  | 0.10 | 8.35 |
| Potassium sulfate  (K_2_SO_4_) | 0.01 | 6.02 |
|  | 0.05 | 6.57 |
|  | 0.10 | 6.16 |
| Sodium pyrophosphate  (Na_4_P_2_O_7_) | 0.01 | 9.66 |
|  | 0.05 | 9.60 |
|  | 0.10 | 9.61 |
| Sodium hydroxide  (NaOH) | 0.01 | 11.13 |
|  | 0.05 | 12.36 |
|  | 0.10 | 12.76 |

The final enzyme extraction efficiency was determined for six extractants (Figure S.6). The color of the final extractant from the soil can serve as a quick, visual indicator of overall quality of enzyme solution (Figure S.7). The lysate is a rust-red color associated with the heme-containing chlorite dismutase. Final enzyme solutions that have no color had less humic substances but no active enzymes.

The visual observation of the final extractant from the soil was also complemented by the UV-visible spectroscopy which provides a quick proxy of dissolved organic carbon (DOC) concentrations. The absorbance at 254nm in the UV spectrum detects aromatic humic substances (Edzwald et al., 1985), while the absorbance at 400nm detects color in the visible spectrum (Wallage and Holden, 2010). The ratio (254nm/400nm) provides a measure of humification (Carter et al., 2012; Peacock et al., 2014; Greenfield et al., 2018), where extractants that contain a greater proportion of humic would have lower 254nm/400nm ratio (Graham et al., 2012). The absorbance values at 254nm and 400nm and the ratio (254nm/400nm) of the final extractant from the soil at different concentrations are shown on Table S.4.

Figure S.6 Average activity recovered at different concentrations of the extractants. The recovery efficiency of enzyme activity decreases with the increase in the concentration of the extractant, except for potassium citrate where there is an increase in recovery from 0.05M to 0.1 M. Experiment was performed with biological triplicates and measurements in duplicate. Error bars are standard deviations.


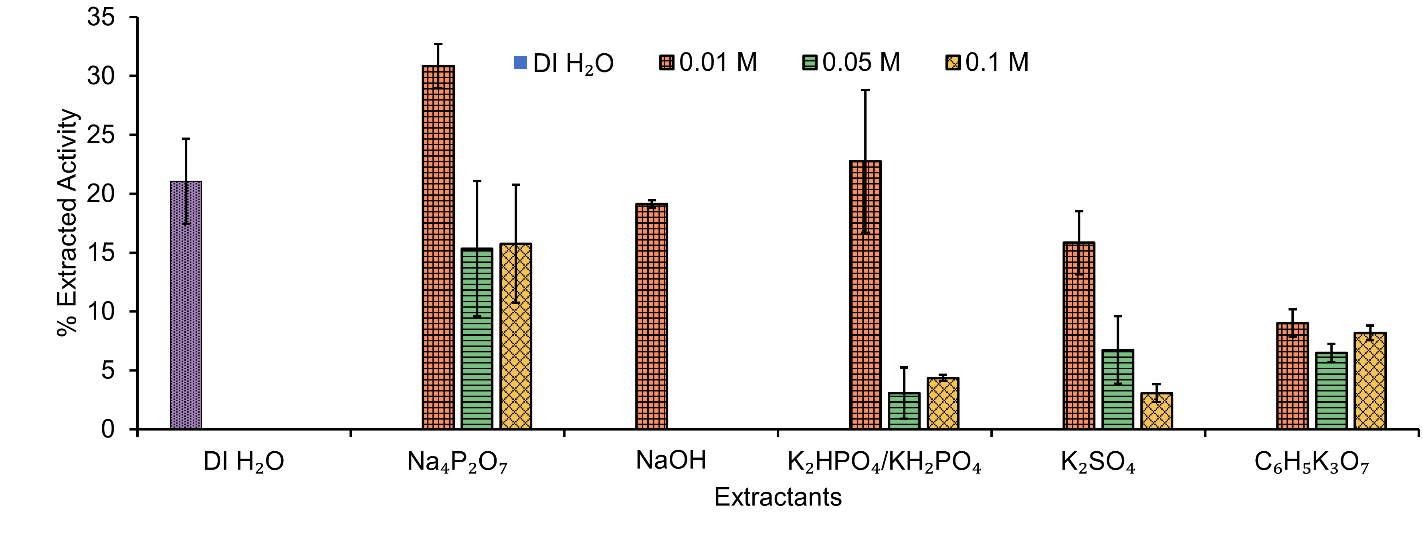


Figure S.7 Images of the supernatants extracted with (a) 0.01 M, (b) 0.05 M, and (c) 0.1 M


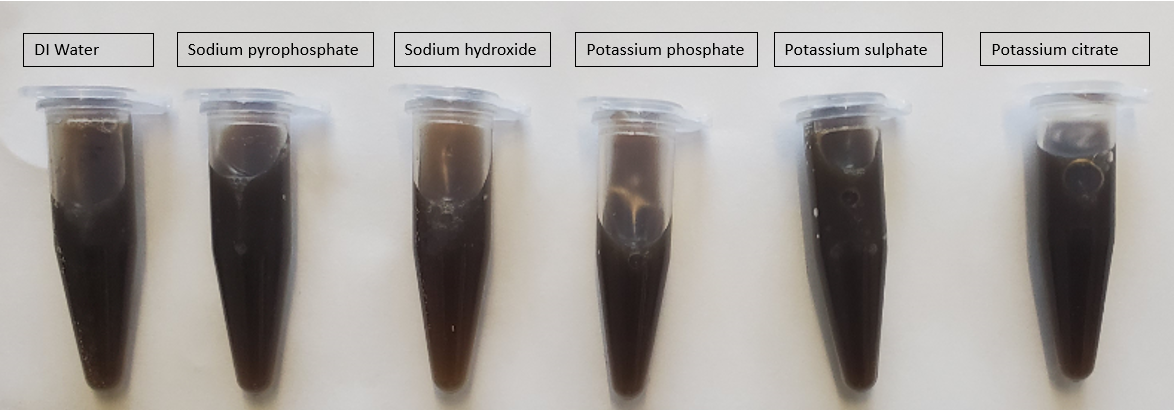

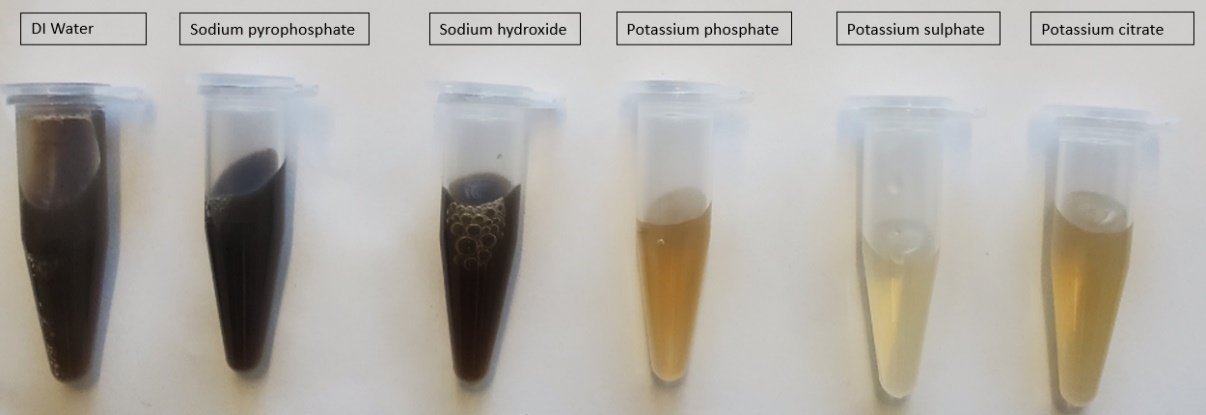

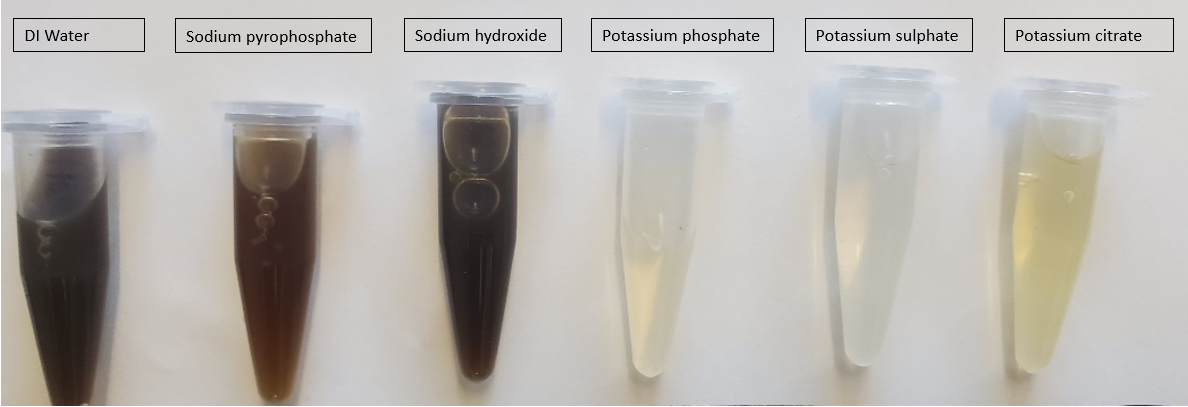


a)

b)

c)

Table S.4: Mean absorbance (AUcm^-1^) of the extractant from the soil at 254nm and 400nm

| **Extractant** | **Concentration (M)** | **254nm** | **400nm** | **254nm/400nm** |
| --- | --- | --- | --- | --- |
| Deionized water | 0 | 5.534 ± 1.238 | 0.797 ± 0.175 | 6.937 ± 0.026 |
| Potassium phosphate | 0.01 | 6.282 ± 0.613 | 1.077 ± 0.237 | 6.064 ± 1.166 |
|  | 0.05 | 0.706 ± 0.315 | 0.061 ± 0.036 | 10.855 ± 0.339 |
|  | 0.1 | 0.408 ± 0.140 | 0.011 ± 0.003 | 61.036 ± 0.179 |
| Potassium citrate | 0.01 | 9.506 ± 1.499 | 1.953 ± 0.669 | 5.341 ± 1.396 |
|  | 0.05 | 0.556 ± 0.146 | 0.042 ± 0.016 | 14.308 ± 2.719 |
|  | 0.1 | 0.458 ± 0.140 | 0.021 ± 0.004 | 36.190 ± 0.259 |
| Potassium sulfate | 0.01 | 7.749 ± 0.426 | 1.240 ± 0.055 | 5.747 ± 0.710 |
|  | 0.05 | 0.437 ± 0.014 | 0.019 ± 0.006 | 20.879 ± 3.203 |
|  | 0.1 | 0.310 ± 0.053 | 0.005 ± 0.000 | 68.278 ± 1.389 |
| Sodium pyrophosphate | 0.01 | 7.804 ± 2.137 | 1.302 ± 0.788 | 7.020 ± 1.772 |
|  | 0.05 | 6.189 ± 0.053 | 0.893 ± 0.025 | 6.932 ± 0.134 |
|  | 0.1 | 3.329 ± 1.621 | 0.423 ± 0.290 | 9.431 ± 2.609 |
| Sodium hydroxide | 0.01 | 7.073 ± 2.383 | 1.291 ± 0.788 | 7.506 ± 2.109 |
|  | 0.05 | 5.378 ± 0.204 | 0.637 ± 0.021 | 8.632 ± 0.276 |
|  | 0.1 | 5.063 ± 0.020 | 0.588 ± 0.054 | 8.690 ± 0.835 |

Running and analyzing the protein gel may provide complimentary information if the subunits of perchlorate reductase; PcrA (110kDa) and PcrB(40kDa), which are involved in perchlorate reduction, are present in the extractants. However, the co-extraction of humic substances with the enzymes may also obscure the protein band in the gel (Matsumoto et al., 2000). Sodium pyrophosphate (0.1M) extractant after centrifugation minimized extraction of humic substances and bands corresponding to the proteins of interest were visualized on the gel (Figure S.8). Potassium salts lanes, however, show neither the protein bands nor the obscuring effects of the humic bands.

Figure S.8 The symbol (1) indicates samples before centrifugation. The symbol (2) indicates samples after centrifugation. The samples before centrifugation for all extractants and for sodium hydroxide after centrifugation appear to have the smearing likely caused by the co-extraction of the humic substances. The smearing makes proper protein size determination difficult. The samples after centrifugation for potassium sulfate, potassium citrate, and potassium phosphate show no protein bands; however, for sodium pyrophosphate protein bands appear to be resolved well. The PcrA, and PcrB, with an approximate molecular weight of 110kDa and 40kDa, are protein subunits of perchlorate reductase involved in the degradation of perchlorate


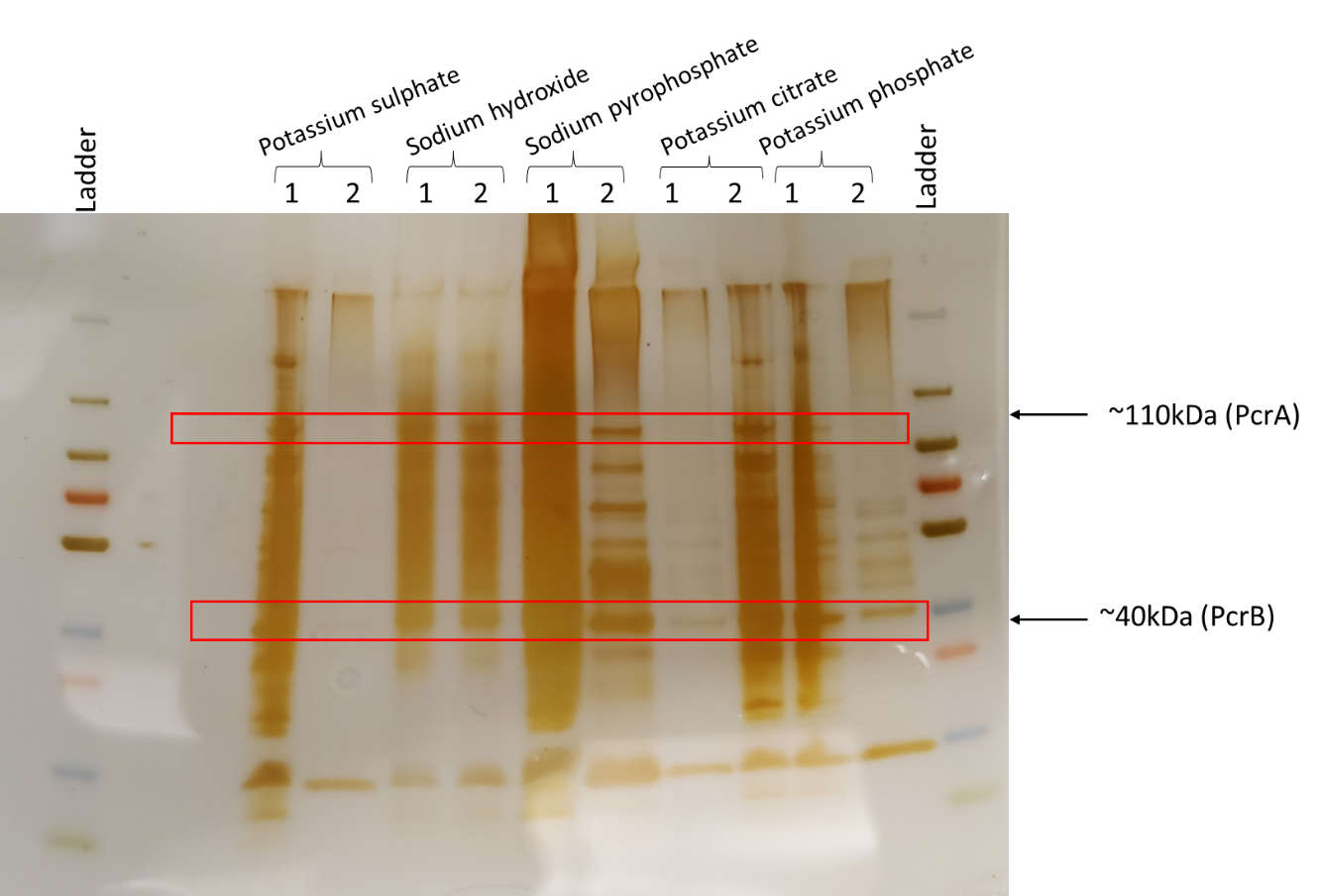


# References

Carter, H.T., Tipping, E., Koprivnjak, J.-F., Miller, M.P., Cookson, B., Hamilton-Taylor, J., 2012. Freshwater DOM quantity and quality from a two-component model of UV absorbance. Water Res. 46, 4532-4542.

Del Pozo, M.V., Martínez-Martínez, M., Ferrer, M., 2014. Protein Extraction from Contaminated Soils and Sediments, In: McGenity, T.J., Timmis, K.N., Nogales, B. (Eds.), Hydrocarbon and Lipid Microbiology Protocols. Springer Berlin Heidelberg, Berlin, Heidelberg, pp. 215-231.

Edzwald, J.K., Becker, W.C., Wattier, K.L., 1985. Surrogate parameters for monitoring organic matter and THM precursors. J. Am. Water Works Assoc. 77, 122-132.

Graham, M.C., Gavin, K.G., Kirika, A., Farmer, J.G., 2012. Processes controlling manganese distributions and associations in organic-rich freshwater aquatic systems: the example of Loch Bradan, Scotland. Sci. Total Environ. 424, 239-250.

Greenfield, L.M., Hill, P.W., Paterson, E., Baggs, E.M., Jones, D.L., 2018. Methodological bias associated with soluble protein recovery from soil. Sci. Rep. 8, 1-6.

Hutchison, J.M., Guest, J.S., Zilles, J.L., 2017. Evaluating the development of biocatalytic technology for the targeted removal of perchlorate from drinking water. Environ. Sci. Technol. 51, 7178-7186.

Hutchison, J.M., Poust, S.K., Kumar, M., Cropek, D.M., MacAllister, I.E., Arnett, C.M., Zilles, J.L., 2013. Perchlorate reduction using free and encapsulated *Azospira oryzae* enzymes. Environ. Sci. Technol. 47, 9934-9941.

Hutchison, J.M., Zilles, J.L., 2015. Biocatalytic perchlorate reduction: kinetics and effects of groundwater characteristics. Environ. Sci. Water Res. Technol. 1, 913-921.

Hutchison, J.M., Zilles, J.L., 2018. Biocatalytic removal of perchlorate and nitrate in ion-exchange waste brine. Environ. Sci. Water Res. Technol. 4, 1181-1189.

Liu, J., Li, J., Feng, L., Cao, H., Cui, Z., 2010. An improved method for extracting bacteria from soil for high molecular weight DNA recovery and BAC library construction. J. Microbiol. 48, 728-733.

Matsumoto, S., Ae, N., Yamagata, M., 2000. Extraction of mineralizable organic nitrogen from soils by a neutral phosphate buffer solution. Soil Biol. Biochem. 32, 1293-1299.

McClatchey, K.D., 2002. Clinical laboratory medicine. Lippincott Williams & Wilkins.

Peacock, M., Evans, C.D., Fenner, N., Freeman, C., Gough, R., Jones, T.G., Lebron, I., 2014. UV-visible absorbance spectroscopy as a proxy for peatland dissolved organic carbon (DOC) quantity and quality: considerations on wavelength and absorbance degradation. Environ. Sci.: Process. Impacts 16, 1445-1461.

Storey, K.B., Storey, J.M., 1988. Freeze tolerance in animals. Physiol. Rev. 68, 27-84.

Tartaglia, M., Bastida, F., Sciarrillo, R., Guarino, C., 2020. Soil Metaproteomics for the Study of the Relationships Between Microorganisms and Plants: A Review of Extraction Protocols and Ecological Insights. Int. J. Mol. Sci. 21, 8455.

Thorneley, R.N., 1974. A convenient electrochemical preparation of reduced methyl viologen and a kinetic study of the reaction with oxygen using an anaerobic stopped-flow apparatus. Biochim. Biophys. Acta - Bioenerg. 333, 487-496.

van Loosdrecht, M.C., Nielsen, P.H., Lopez-Vazquez, C.M., Brdjanovic, D., 2016. Experimental methods in wastewater treatment. IWA publishing.

Wallage, Z., Holden, J., 2010. Spatial and temporal variability in the relationship between water colour and dissolved organic carbon in blanket peat pore waters. Sci. Total Environ. 408, 6235-6242.

Wolkers, W.F., Balasubramanian, S.K., Ongstad, E.L., Zec, H.C., Bischof, J.C., 2007. Effects of freezing on membranes and proteins in LNCaP prostate tumor cells. Biochim. Biophys. Acta - Biomembr. 1768, 728-736.
